# Bactericidal Effect of Silver Nanoparticles on Intramacrophage *Brucella abortus 544*

**DOI:** 10.5812/jjm.9039

**Published:** 2014-03-01

**Authors:** Hamed Alizadeh, Mojtaba Salouti, Reza Shapouri

**Affiliations:** 1 Young Researchers and Elite Club, Zanjan Branch, Islamic Azad University, Zanjan, IR Iran; 2 Biology Research Center, Zanjan Branch, Islamic Azad University, Zanjan, IR Iran

**Keywords:** *Brucella abortus*, Nanoparticles, Macrophage, Antimicrobial

## Abstract

**Background:**

Brucellosis is an infectious disease that is caused by *Brucella *spp. As *Brucella *spp. are intramacrophage pathogens, the treatment of this infection is very difficult. On the other hand, due to the side effects of the brucellosis treatment regime, it is necessary to find new antimicrobial agents against it.

**Objectives:**

The aim of this study was to investigate the antimicrobial effect of silver nanoparticles against *Brucella abortus 544* in the intramacrophage condition.

**Materials and Methods:**

The antimicrobial effect of silver nanoparticles was determined by an agar well diffusion method. The minimum inhibitory concentration (MIC) and minimum bactericidal concentration (MBC) of silver nanoparticles against *B. abortus* 544 were determined by a broth macrodilution method. The effect of time on the antimicrobial activity of silver nanoparticles was analyzed. The effect of silver nanoparticles on the intramacrophage survival of *B. abortus 544* was studied on mice peritoneal macrophages.

**Results:**

The well diffusion agar study showed that silver nanoparticles have an antimicrobial effect on *B. abortus 544*. The MIC and MBC of silver nanoparticles against *B. abortus* 544 were; 6 ppm and 8 ppm, respectively. The silver nanoparticles showed antibacterial effects within 40 minutes. The results of the macrophage culture indicated that silver nanoparticles have antibacterial activity against intramacrophage* B. abortus 544,* and the highest efficiency was observed at a concentration of 8-10 ppm of silver nanoparticles.

**Conclusions:**

The results showed that silver nanoparticles have an antimicrobial effect against intramacrophage *B. abortus 544*.

## 1. Background

Brucellosis is an infectious disease caused by *Brucella *spp. Treatment for brucellosis remains controversial and requires prolonged therapy with at least two antibacterial agents, such as doxycycline and rifampin. *Brucella* species are Gram-negative bacteria that cause disease in humans and other mammals, such as sheep, goats and cattle. *Brucella abortus* remains a major cause of morbidity in humans and domestic animals. After invasion of the lymphoid system, the bacteria develop within mononuclear phagocytes, and the infected cells play a crucial role in the dissemination of the bacteria to specific body organs, such as; spleen, brain, heart, and bones (1-3). Animal feed contamination results in abortion and infertility, and the human infection is known as undulant or Malta fever (4).

People generally have direct contact with brucellosis through infected animals or their products, by accidental self-inoculation with animal vaccine strains, or as a result of laboratory accidents. Human infection presents as a prolonged debilitating febrile illness. *Brucellae *produce chronic and often lifelong infections in their natural hosts (5). The *Brucella *genus is comprised of six recognized species based on host specificity. While infections caused by all six species occur at least sporadically in the United States, the greatest economic impact results from bovine brucellosis and it is caused by *B. abortus*. The infection decreases reproductive efficiency, mainly through abortion. The disease has also led to restrictions in the international and interstate movement of animals.

*B. abortus *is the main cause of nearly all cattle abortions that result from brucellosis. *B. melitensis* and *B. suis* infect cattle, which spread within herds, but rarely cause abortions (6). The persistent nature of these infections is predominantly due to the ability of these bacteria to maintain intracellular residence in host macrophages. Thus, the virulence and chronic infections of *Brucella* species are thought to be due to their ability to escape killing mechanisms within macrophages, such as lysosomal enzymes and products of the oxidative burst (1). Treatment of brucellosis with a single antibiotic regime is not recommended due to high rates of relapse. However, a clear optimal dual therapy has not been agreed upon (7).

Different studies have shown the bactericidal effect of silver nanoparticles in Gram-negative and Gram-positive bacteria (8, 9). Morones et al. defined the antibacterial activity of silver nanoparticles in four types of Gram-negative bacteria, including;* Escherichia coli*, *Vibrio cholera*, *Pseudomonas aeruginosa*, and *Salmonella typhi, *which suggests that silver nanoparticles attach to the surface of the cell membrane, disturb its function and penetrate into the bacteria (10). Other researchers have reported a similar antibacterial activity in Gram-positive bacteria, such as; *Bacillus subtilis*, *Staphylococcus aureus*, and *Enterococcus faecalis *(11-13). Silver nanoparticles have also been found to have antibacterial activity against some drug-resistant bacteria, such as;* E. coli*, *P. aeruginosa,* and *S. aureus *(14, 15). However, the antimicrobial effect of silver nanoparticles on *B. abortus 544* and intramacrophage *B. abortus* 544 have not been studied so far.

## 2. Objectives

The aim of this study was to investigate the antimicrobial effect of silver nanoparticles on intramacrophage *B. abortus 544*.

## 3. Materials and Methods

### 3.1. Materials and Media

*B. abortus 544* strain was obtained from the Department of Bacterial Vaccine and Antigen Production of the Pasteur Institute, Iran. Silver nanoparticles were obtained from the NANOCIDE Company, Iran with a size of 3-18 nm and a concentration of 4000 ppm. All microbial media were obtained from Merck, Germany.

### 3.2. Antimicrobial Activity of Silver Nanoparticles on *B. abortus* 544

Muller-Hinton agar was supplemented with 1% sheep hemoglobin (Hb), then 5 mm diameter wells were prepared. Next, 1.5×10^8 ^CFU/mL of *B. abortus 544* suspension was spread on the plates with a sterile swab. Then, the wells were loaded with different dilutions of silver nanoparticles (70 μL of 2, 5, 10, 20, 50, 100, 250, 500, 1000 and 2000 ppm of silver nanoparticles). The plates were incubated at 37^o^C under 7-10% CO_2_ for 72 hours. The diameter of the growth inhibition zone was measured by a ruler (16, 17). The tests were performed in triplicate.

### 3.3. MIC and MBC Determination

Serial dilutions of silver nanoparticles (1, 2, 5, 10, 20, 50, 100, 250, 500, 1000 and 2000 ppm) were prepared in a Muller-Hinton broth supplemented with 1% of sheep blood. Then, 5×10^5 ^CFU/mL of *B. abortus* 544 suspension was added to each tube and incubated at 37^˚^C under 7-10% CO_2_ for 72 hours. Next, the tubes were examined for turbidity, which indicates the growth of microorganisms. The lowest concentration of silver nanoparticles that inhibits the growth of *B. abortus* 544 was designated as the minimum inhibitory concentration (MIC). To measure the minimum bactericidal concentration (MBC) of silver nanoparticles, 0.1 mL of inoculums from each tube were sub-cultured on Muller-Hinton agar plates supplemented with 1% sheep blood (18). The number of colonies on the agar were counted under the same conditions after a72-hour incubation period, and then compared with the number of CFU/mL in the original inoculums. The lowest concentration of silver nanoparticles that is capable of killing the bacteria was determined as the MBC. The tests were carried out three times.

### 3.4. Effect of Time on Antimicrobial Action

The MBC concentration of silver nanoparticles was prepared in Muller-Hinton broth, supplemented with 1% sheep blood, in a tube. Then, 5×10^5 ^CFU/mL of *B.* *abortus 544* suspension was added to the tube and incubated at 37^˚^C under 7-10% CO_2_. At 0 min, 20 min, 40 min, 1 h, 2 h, 4 h, 6 h and 24 h, after culturing, 0.1 mL of inoculums from the tube were sub-cultured on Muller-Hinton agar plates supplemented with 1% sheep blood. Meanwhile, at the same time a tube (without silver nanoparticles) was used as a negative control, containing the same amount of media, and bacteria. The growth or absence of growth of *B. abortus* *544* after 72 hours of incubation under the same conditions was investigated in order to find the shortest time required to kill the bacteria (18). The tests were performed in triplicate.

### 3.5. Effect of Silver Nanoparticles on Macrophages

Thioglycollate-elicited peritoneal exudate cells were obtained from 6-8 week old female BALB/C mice. First, 1 mL thioglycollate broth (4.05 g/100 mL) (Difco, USA) was injected intraperitoneally. Four days later, 5 mL of; RPMI 1640, Q-Lab, USA, medium, with 10% heat-inactivated fetal calf serum (FCS) was injected intraperitoneally along with 5 unit of heparin. Then, the lavage of the peritoneal cavity was vacuumed using a sterile syringe and the cells were washed twice and resuspended in RPMI 1640 with 10% FCS and placed in 96 well polystyrene micro titer plates containing 0.27×10^4^ macrophages per well. The plates were incubated for 2 h at 37^˚^C in 5% CO_2_. Next, the supernatant was aspirated and the adherent monolayer was washed three times with the RPMI 1640 medium. To determine the effect of silver nanoparticles on the viability of macrophages, murine peritoneal macrophages were tested in vitro at different concentrations of silver nanoparticles for 24 h. Peritoneal exudate cells were counted for in vitro viability using a dye exclusion test with trypan blue, before and after 24 h of macrophage incubation with different dilutions of silver nanoparticles (12, 10, 8, 6, 4 and 2 ppm). Normal saline was used as a negative control (18). The tests were carried out three times.

### 3.6. Intramacrophage Activity of Silver Nanoparticles

*B. abortus 544* was cultured at 37^˚^C in *Brucella* agar in 7-10% CO_2 _for 48 h, then resuspended in phosphate-buffer saline (PBS), washed and resuspended in the same buffer. Next, a bacterial suspension (5×10^5 ^CFU/mL) was added to the peritoneal macrophages and incubated for 2 h under the same conditions, allowing the macrophages to ingest the *B. abortus* 544. A solution of 50 μg/mL gentamicin was added and the microplates were incubated for 1 h to kill the extracellular bacteria. Next, the monolayer was washed three times with the RPMI 1640 media and various dilutions of silver nanoparticles (less than 6 ppm) were added. Three wells were used as controls and treated with sterile normal saline instead of silver nanoparticles. After a 24-h incubation, Triton X-100 was used to lysis the macrophages. Then, the number of CFUs in the lysate were determined by serial dilutions and plated on *Brucella* agar. Normal saline was used as a negative control (18). The tests were performed in triplicate.

### 3.7. Statistical Analysis

The results were analyzed by a one way ANOVA test using SPSS (version 18) software (Illinois, USA). P value of 0.05 was considered statistically significant. All of the tests were in triplicate.

## 4. Results

### 4.1. Antibacterial Activity of Silver Nanoparticles

**Figure 1.**
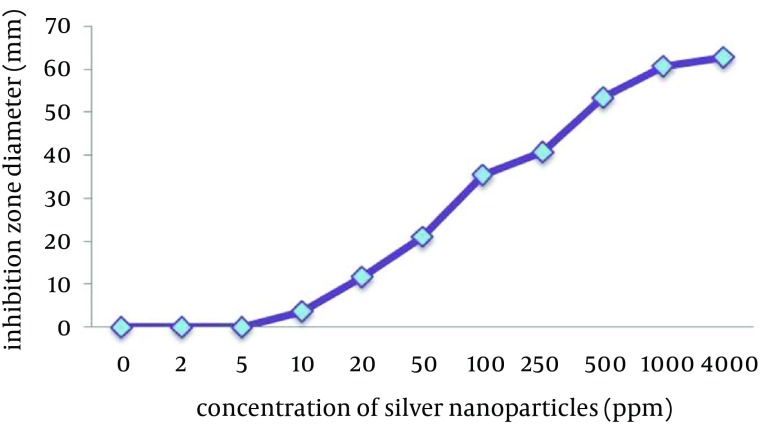
Effect of Different Concentrations of Silver Nanoparticles on *B. abortus 544*

The agar-well diffusion method showed that *B. abortus 544* was sensitive to silver nanoparticles. Moreover, the results showed that silver nanoparticles have an antimicrobial effect against *B. abortus 544* at low concentrations (Figure 1). 

### 4.2. MIC and MBC Determination

The results showed that the MIC and MBC of silver nanoparticles, determined by the broth macrodilution method *on B. abortus 544,* was 6 ppm and 8 ppm, respectively.

### 4.3. Effect of Time on Antimicrobial Activity

The study on the time effect of silver nanoparticles’ antibacterial activity against *B. abortus 544,* showed that it had an antibacterial effect within 40 minutes. In the control tubes (without silver nanoparticles) the growth of *B. abortus 544* on the sub-culture in the control tubes was observed at all of the times.

### 4.4. Effect of Silver Nanoparticles on Macrophages

The viability of the macrophages was determined at different dilutions of silver nanoparticles before and 24 h after incubation. The results indicated that silver nanoparticle concentrations greater than 6 ppm had an inhibitory effect on peritoneal macrophages, while this effect was not observed at lower concentrations (Table 1). Therefore, in this study we used concentrations less than 6 ppm in order to study the effect of silver nanoparticles on intramacrophage *B. abortus* *544*.

**Table 1.** Viability of Macrophages at 0 and 24 Hours After Treatment With Silver Nanoparticles

Concentration of Silver Nanoparticles	Percentage of Viable Macrophages, Mean ± SD
**Lack of silver nanoparticles at 0 h**	95 ± 3
**Normal saline (control) after 24 h**	92 ± 4
**12 ppm after 24 h**	63 ± 4^a^
**10 ppm after 24 h**	70 ± 5^a^
**8 ppm after 24 h**	85 ± 4 ^a^
**6 ppm after 24 h**	89 ± 0
**4 ppm after 24 h**	90 ± 5
**2 ppm after 24 h**	93 ± 6

^a^ Indicates a significant difference compared with the control group. Normal saline was used as a negative control.

### 4.5. Effect of Silver Nanoparticles on Intramacrophage B. abortus 544

The results showed a significant decrease in the number of colonies forming on the test plates, compared with the control group (Table 2). 

**Table 2.** Numberof Intramacrophage *Brucella *CFU/mL of Lysate by Plating

Silver Nanoparticles Concentration	Number of Bacteria, Mean ±SD
**Control (normal saline)**	3360 ± 825
**10 ppm**	6.4 ± 2
**8 ppm**	12.8 ± 4
**6 ppm**	83.2 ± 12
**4 ppm**	291.2 ± 33

## 5. Discussion

Nowadays, people all over the world try to avoid chronic stress, pollution and synthetic drugs. It is well documented that the number of pathogenic bacteria resistant to current antibiotics has increased significantly, and infections caused by resistant strains of bacteria pose serious clinical problems (1).

*Brucella *spp. is capable of invading and surviving in both phagocytic and non-phagocytic host cells. Macrophages, dendritic cells (DCs), and trophoblasts are the major target cells for *Brucellae*. This is according to the clinical manifestations of brucellosis in experimental and natural hosts, which are characterized by the detection of persistent infectious agents in lymphoid tissues and inflammatory lesions in the reproductive tracts of pregnant females. Bacterial entrance, survival and replication have been intensively investigated in phagocytes, but these mechanisms are poorly characterized in trophoblasts, which represents an important gap in our understanding about the disease and transmission among its natural hosts. In order to reach its target cells, *Brucella *needs to pass through the mucosal barriers of the respiratory, genitourinary or digestive tracts, where it undergoes phagocytosis by resident macrophages and DCs, resulting in dissemination of the organism to lymphoid and reproductive organs (19). *Brucella *spp. are relatively sensitive to a wide range of antibacterial agents, but in a single drug regimen, relapses are common. *Brucella* spp. is facultative intracellular bacteria and they can survive within phagocyte cells. The intracellular survival of *Brucella* is the most important factor in the virulence of this bacteria and its pathogenicity (20).

The antimicrobial effects of silver nanoparticles on *B. abortus* 544 and macrophages have not been studied so far. In this study we found that silver nanoparticles have an antimicrobial effect on intramacrophage *B. abortus 544*. The MBC, MIC and tests at 2 ppm and 1 ppm silver nanoparticle concentrations have demonstrated a significant effect on the elimination of intramacrophage *B. abortus* 544 over a period of 24 hours. Therefore, silver nanoparticles are able to penetrate into the macrophage cells and kill *B. abortus 544,* in addition to their broth dilution and agar-well diffusion antimicrobial activity. It can be concluded that silver nanoparticles may be useful for the treatment of diseases, caused by intracellular microorganisms such as *Brucella*, while there are some antibiotics that are not effective in vivo conditions.

The results showed that silver nanoparticles can kill *B. abortus 544 *at low concentrations. The antimicrobial activity of these particles depends on the concentration of silver nanoparticles, and by increasing silver nanoparticle concentrations, the bactericidal activity will also increase. A previous study on the effect of time on silver nanoparticle antibacterial activity showed that silver nanoparticles can kill *B. abortus* *544* within 40 minutes of culturing. This result showed that silver nanoparticles have a very fast antimicrobial effect on intramacrophage *B. abortus 544*. This feature can be useful in the rapid control of *Brucella* infections. These results are in agreement with a study by Al-Mariri et al., who reported that *Cinnamom umverum* volatile oil had antimicrobial activity against *B. abortus* *544* (1) and it was also in according with the results of Shapouri et al. who concluded that hops extracts had intramacrophage antimicrobial effects on *B. abortus* and *B. melitensis *(17). The results of this present study showed that silver nanoparticles have an antimicrobial effect against intramacrophage* B. abortus* 544; therefore, it can be useful in the treatment of brucellosis. The authors suggest the evaluation of antibacterial effect of other metal nanoparticles on intramacrophage survival of *Brucella* bacteria.
